# Awareness as observational heterarchy

**DOI:** 10.3389/fpsyg.2013.00686

**Published:** 2013-10-01

**Authors:** Kohei Sonoda, Kentaro Kodama, Yukio-Pegio Gunji

**Affiliations:** ^1^Department of Education, Faculty of Education, Shiga UniversityOtsu, Japan; ^2^Department of Informatics, School of Multidisciplinary Science, The Graduate University for Advanced StudiesChiyoda-ku, Japan; ^3^Department of Earth and Planetary Science, Faculty of Science, Kobe UniversityKobe, Japan; ^4^The Unconventional Computing Centre, University of the West EnglandBristol, UK

**Keywords:** prediction, postdiction, internal measurement, heterarchy, awareness, emergence, wholeness

## Abstract

Libet et al. (1983) revealed that brain activity precedes conscious intention. For convenience in this study, we divide brain activity into two parts: a conscious field (CF) and an unconscious field (UF). Most studies have assumed a comparator mechanism or an illusion of CF and discuss the difference of prediction and postdiction. We propose that problems to be discussed here are a twisted sense of agency between CF and UF, and another definitions of prediction and postdiction in a mediation process for the twist. This study specifically examines the definitions throughout an observational heterarchy model based on internal measurement. The nature of agency must be *emergence* that involves observational heterarchy. Consequently, awareness involves processes having duality in the sense that it is always open to the world (postdiction) and that it also maintains self robustly (prediction).

## Introduction

Libet et al. (1983) reported mounting brain activity related to a resultant action for approximately three hundred milliseconds before subjects reported their first awareness of a conscious intention to act. In other words, conscious decisions to act were clearly preceded by an unconscious buildup of electrical charge within the brain. This buildup came to be called readiness potential (RP). Such a division between the conscious field (CF) and the unconscious field (UF) can be found in postscripts of intention in experiments^1^. Stimulating particular brain regions led to reactions of particular body parts without a subject's own intention (Delgado, 1969; Penfield, 1975). Consequently, one attributes actions executed by others (not one's own actions) to one's intention (Wegner et al., 2004). These results are explained as below. After an efferent copy of an initial motor command is generated and simulated, it is compared with afferent information from sensory feedback as a result of actual movement. In the case of congruence between efferent and afferent information, it is said that one experiences a sense of agency for the movement (e.g., Gallagher, 2000; Synofzik et al., 2008a,b). Here, these studies are based on the idea of a hierarchy comprising a higher monitoring part and a lower part executing actual movement. Results reported for an apparent mental causal path (Wegner and Wheatley, 1999; Wegner, 2003) show that that conscious will is subject to unconscious will and the comparator model (e.g., Wolpert et al., 1995; Frith et al., 2000a). However, the system presumed in those studies is hierarchical, not heterarchical (McCulloch, 1945).

**Figure 1:**
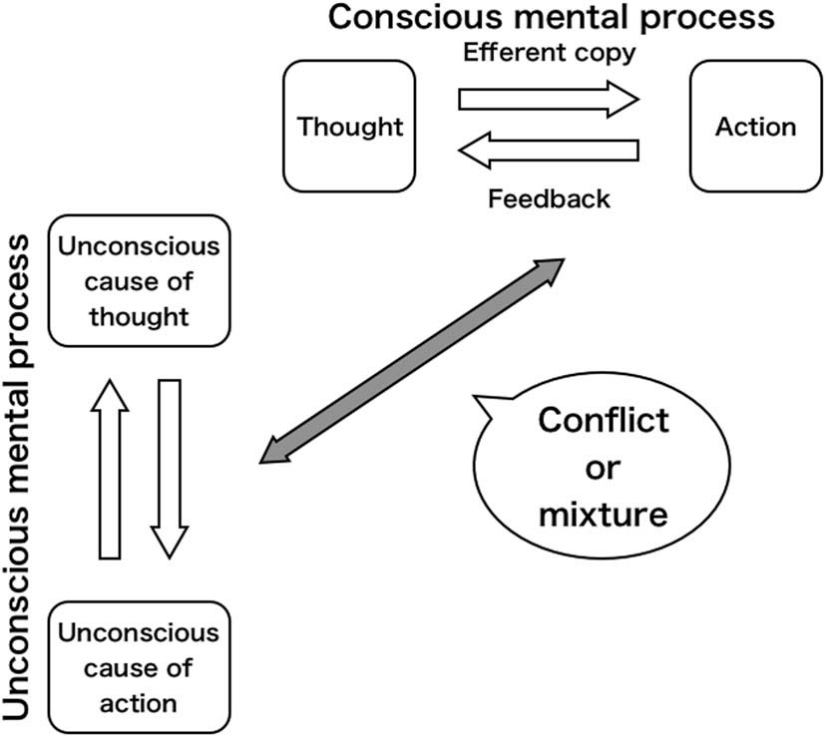
**Duality of mental process**.

Most previous studies have specifically investigated ways of mechanism comparing conscious intention with movement result. When expressing the comparing mechanism as a pair of thought–action, the pair is usually assumed as that of CF in those earlier studies. We raise a question of whether a pair of thought–action will be dual in brain. It means duality of the pair in CF and UF (Figure 1). Considering that the area playing a role of conscious will is just a part of brain, and that it is separate from areas generating actual motor command. We can accept some kind of independence between CF and UF, and assume dual pairs of thought–action (dual operating systems). Gunji (2013) showed that the area comparing an efferent copy with movement results is not merely a monitoring area but rather CF, and the RP area (UF) plays a role of execution of specific movements preceding CF. Comparing CF, UF is absolutely *others* in the brain. Gunji (2013) argued that an origin of voluntariness comes from such a twisted feeling of operation. One has a sense of being operated by *others* in the brain. Nevertheless, that person finds out that the *other* is himself. When we have a feeling of operation, we also face a difficulty of self-reference that “I operate on me.” “I” operating (subjective *self*) and “Me” operated (objective *self*) are strictly different in status. Thus, “I operate on me” is fragile. Consequently, “*others* in the brain (UF) operate on me” is a stronger keynote than “I (CF) operate on me.” Moreover, it should be found that the *other* is just “I” (myself). We argue that the twist is an origin of the sense of agency (SoA). Then, the *other* in the brain can become not only “myself” but also “someone unknown” or “you” just in front of me.

What is important here is the twisted viewpoint of accepting a mixture of “I” (CF) and “the *other*” (UF) while assuming some mutual independence^2^. As described above, the twist can become the origin of voluntariness, but simultaneously engender the crisis of a system such as self in autism or integration disorder syndrome (e.g., Frith, 1989; Frith et al., 2000b). Therefore, we can describe a schematic model of conflict = mediation between CF and UF or “I” and “the *other*” (Figure 1). Then we would suggest that prediction and postdiction could be identified in a process of mediation. We presume that aspects of prediction and postdiction do not appear in previous studies (e.g., Blakemore et al., 2002; Bays et al., 2006; Synofzik et al., 2013). Those studies examined only problems in comparing motor intention with movement result in CF. We do not specifically examine such a simple problem on the comparison mechanism in CF. Beyond it we would rather specifically examine the conflict between two operating systems: CF and UF. Consequently, we aim to redefine prediction and postdiction from the conflict in this study. In the conflict, the difference between prediction and postdiction is a gap separating “I” of CF and *others* in brain of UF. The gap is just the origin of voluntariness. Prediction stands for the aspect of equalizing “I” and *others* in brain by erasing the gap. However, postdiction means the aspect of materializing the gap as “someone” by being open to the world. In the next section, we dissert these aspects in detail through an observational heterarchical model, with a dynamic hierarchy including a latent mixture of levels.

## Agency and emergence

What is the nature of agency? It must be that of emergence. Our argument expressed in this paper is that the nature of agency is that of emergence. *Other* (UF) operates on *me* (CF). Furthermore, I find that *the other* is *I*. This characteristic is the very emergence of agency. However, most current discussions depend on the comparator model (Frith et al., 2000b) that agency derives from a mechanism, and the judgment problem of whether it occurs before an event or after: roughly speaking, we are machines with agency and only judge events' timing, which sometimes reveals errors. The model cannot explain a vicarious agency with no efferent copy in which a person feels that one is doing something despite actually doing nothing himself (Wegner et al., 2004). A feeling of doing is only illusion if it is not accurate (Wegner, 2002). Herein, we can identify a dichotomy between the two: mechanism or illusion (Table 1). The problem is not abnormality or illusion of agency but normal agency that we feel in daily life. The daily life agency, a feeling that “I” operate on me, is not fundamental (mechanism). Then we obtain from the nature of emergence that *the other is I*^3^. Consequently, the salient difficulty is not a lack of experimental evidence but the concept of *emergence*.

**Table 1:** **Dichotomies on the notion of awareness**.

Mechanism	Hierarchy	Determinism
Illusion	Heterarchy	Vitalism

As described in this paper, we attempt to describe the nature of agency using the notion of “observational heterarchy” (Gunji and Kamiura, 2003, 2004). In this section, we introduce notions of emergence. In the subsequent section, we also discuss this point in light of the notions of “hierarchy” (Salthe, 2012) and “heterarchy” (Stark, 1999). In the third section, we introduce the notion of observational heterarchy.

Notions of emergence have been discussed for a long time. There are many definitions of emergence (O'Connor and Wong, 2012). We can identify some kinds of hierarchical structures assume under the various notions (Barabási and Albert, 1999; Odum and Barrett, 2004; Postle, 2006). We briefly define emergent phenomenon as macroscopic patterns running through underlying microscopic interactions. For example, when we observed a population that has a new novel ability that the others of the same species do not have, we call that observation one of an emergent phenomenon. For explanations of such an emergent phenomenon, there are many discussions in philosophy (e.g., Kim, 1999; Bedau, 2008; Bitbol, 2012). However, these philosophical discussions are beyond the scope of this paper. Therefore, we only show a model of observational heterarchy as one of models of emergent phenomena in this paper^4^. And we discuss that the notions of hierarchy and heterarchy cannot be models of emergence and thus comparator model = mechanism (Frith et al., 2000b) or apparent mental causation = illusion (Wegner, 2002) cannot explain the nature of agency (Table 1).

## Hierarchy and heterarchy

**Figure 2:**
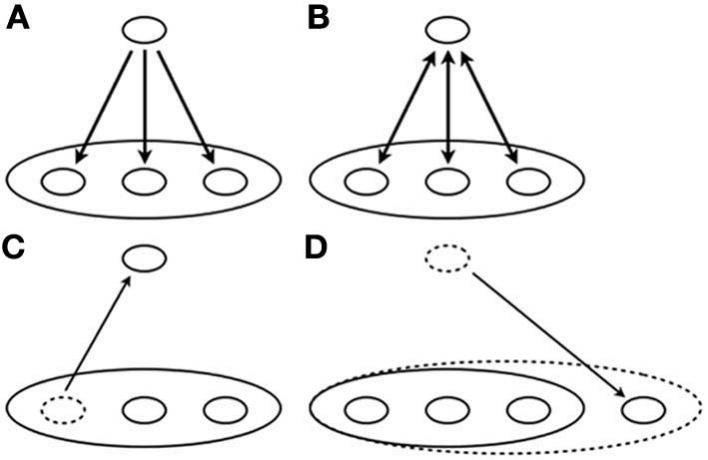
**Schematic diagram of (A) hierarchy, (B) heterarchy, (C) observational heterarchy with compression effect, and (D) observational heterarchy with extension effect**.

First, we define the notions of hierarchy and heterarchy in this paper. Hierarchy is identifiable as some kind of order structure of a company (Figure 2A). Cladograms of taxonomy are also familiar^5^. Therefore hierarchy is definable as a partial ordered set (POS)^6^. Heterarchy is a dynamical hierarchy including of a mixture of levels (Figure 2B). Although heterarchy is apparently consistent, as in some discussions (McCulloch, 1945; Stark, 1999; Norman et al., 2010), it is inconsistent in the strict sense of the word (Salthe, 2012). Consequently, it cannot have its formal expression attributable to its logical flaw for the mixture as described in the discussion presented below.

The wholeness^7^ that the notion of hierarchy invariably depends on is “transcendental wholeness” (Gunji, 2006)^8^. Transcendental wholeness is a privileged concept that differs from other concepts because of the point that it is not permitted to have an extent-perspective^9^,^10^. This wholeness seals the discussion of interaction between parts and a whole. Even if we discuss a hierarchical world (system), we cannot address a variation of the world (emergence). The wholeness of set theory is this transcendental one. This notion avoids Russell's paradox^11^ and removes inter-level interaction^12^. We can also identify the removal of the mixture of levels from the notions of hierarchy (Salthe, 1985, 2012). Consequently, the transcendental wholeness corresponds to the concept of hierarchy.

Heterarchy is “contradictory wholeness” (Gunji, 2006). The second wholeness implies a whole consisting of parts while defining the whole as a contraposition to the parts. Furthermore, we obtain a contradiction of the concept. This wholeness appears in Russell's paradox (Whitehead and Russell, 1925). In other words, the second wholeness permits a mixture of levels: the mixture leads to Russell's paradox. Consequently, this wholeness corresponds to the notions of heterarchy that permit the mixture (McCulloch, 1945; Stark, 1999; Norman et al., 2010).

What is the difference between a transcendental wholeness and a contradictory one? It is the restriction of extent-perspective: a mixture of levels. Contradictory wholeness is an unrestrictive version of transcendental wholeness. The difference appears when we examine the “whole” of a description (a system or hierarchy). In Russell's paradox, when we survey the whole of all sets, the difference appears^13^. The difference is latent until we survey the whole of the description of sets. Roughly speaking, it had been latent until Russell found it. Here, we emphasize that the difference between the two notions of wholeness is not limited in mathematics. For the discussions presented above, we correspond hierarchy and heterarchy, respectively, to the comparator model (Frith et al., 2000b) = mechanism, and the apparent mental causation (Wegner and Wheatley, 1999) = illusion. Determinism and vitalism are the same case (Table 1). In understanding of history, we also divide human history into two parts—stable periods and change periods—. We understand it through alternation of the first wholeness and the second wholeness. In such a dichotomy between the two, we can identify the fragile relation between affirmation of the world (stable period) and negation of the world (change period) to ascertain the wholeness of history comprehensively. Here we can expect the key that connects the two notions of wholeness as two different phases that comprise the nature of the world and of our understanding of the world.

## Observational heterarchy

**Figure 3:**
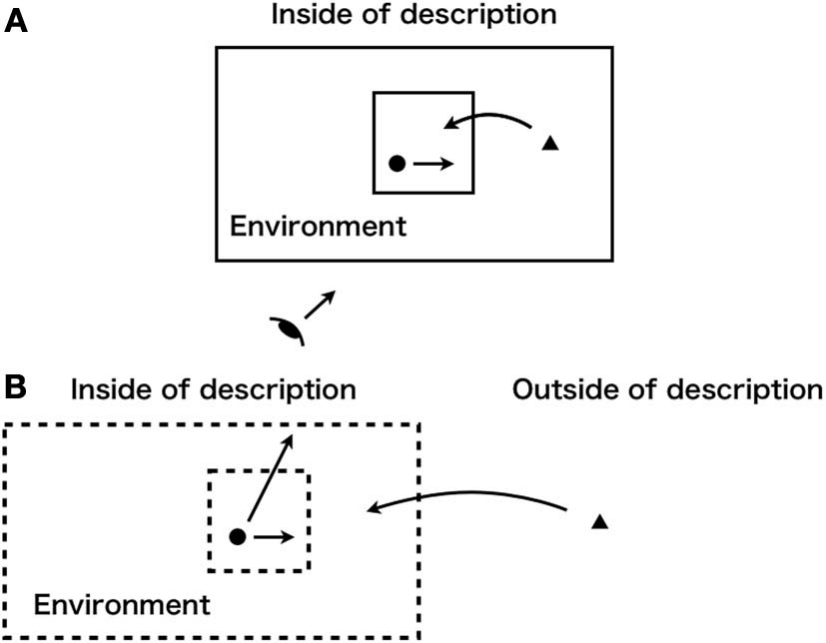
**Observer with partial knowledge (inner square of line or dotted line) is inside of the description and emergent properties originate from somewhere: schematic diagram of (A) transcendental view and (B) internal measurement**.

What should we do about the problem that emergent phenomena are beyond description (the first and second wholeness)? We cannot describe the phenomena. However, we, herein, strive to reveal the nature of emergence. The key to the problem must be reconsideration of the concept of wholeness that description is based on. Description invariably accompanies the notion, but remains outside of it. However, system theories construct models without consideration of this characteristic of description. The models are based on a transcendental viewpoint by which an emergent element (component or one level) derives from inside of the description (Figure 3A). We do not designate this picture as one showing emergence. Therefore, we reconsider the nature of description with internal measurement (Matsuno, 1989; Gunji, 1993; Gunji et al., 1997) in which an emergent element originates from outside of the description (Figure 3B). We express the characteristic by an agent's apparent reference of its description, which seems to lead to a self-referential paradox (without this reference, the transcendental perspective reappears). Moreover, we construct invalidation of the paradox by a frame problem. Nevertheless, the model remains a mere description. Therefore, our construction is a model that implies the nature of emergence. Specifically we use weak duality of intent- and extent-perspectives of a description. In this section, we introduce the notion of observational heterarchy (Gunji and Kamiura, 2003, 2004) as the third wholeness: “weak wholeness” (Gunji, 2006)^14^). This third notion of wholeness connects the other two. In the discussion presented above, the first and second wholeness appear in Russell's paradox: a mixture of levels. Thus, we reconsider this mixture.

Although the notion of heterarchy sounds contradictory, it aims at the nature of emergence: a mixture of levels. Why do we specifically examine the mixture? We do so because it is not limited in the problem of an abstract concept. We can find, in biology, some evidence that we can call not developments but evolutions. Important evidence for it is adaptive mutation (Shapiro, 1997, 2002). Splitting enzymes for sugar are controlled by an operon on DNA. If it switches on, an enzyme is expressed, if it is switched off, then it is not. In the experiment of adaptive mutation, *Escherichia coli* bacteria are cultured in culture media with sugar. The DNA of bacteria is converted not to express the splitting enzyme corresponding to the sugar. The bacteria have difficulty surviving because of the absence of the enzyme, which gives rise to a malfunction of the DNA–protein system. The mutation rate becomes high, and mutation hits the broken gene corresponding to the splitting enzyme for the sugar. Consequently, the bacteria can acquire the ability to use the sugar as energy source. DNA is definable as a higher level than cell interactions corresponding to the wasting state because proteins (enzymes) control the interactions and DNA also control the proteins in the bacteria. For adaptive mutation, the cell interactions affect DNA's behaviors directly, whereas DNA usually controls them through the enzyme. Here we can identify an apparent mixture or interaction of different levels—DNA and cells—in the bacteria. Furthermore, it can be expressed as two processes that do not involve hierarchy or heterarchy. When a malfunction of the DNA–protein system occurs in a focal level, the cell level, the DNA mutation rate becomes high in the upper level: the DNA level (Figure 2C). Consequently, the mutation hits the broken gene in the upper level and the splitting enzyme becomes activated at the focal level (Figure 2D). This image motivates us to consider the notion of observational heterarchy as a robust model for a mixture of levels.

Here we quote the summary of observational heterarchy presented in Gunji and Kamiura (2003) below.

*(1) Heterarchy*^15^ *consists of two levels and inter-level operations. (2) Simultaneous interaction among levels is defined as simultaneous choice that is expressed as a surjective map from a set of one level to a set of inter-level operations. (3) Simultaneous choice implies the collapse of the logical framework; then heterarchy is regarded as a system inheriting logical collapse. (4) Because of the logical collapse, heterarchy gives rise to re-organization of the structure. (5) Heterarchy is not a real entity but it results from the interaction between an object and an observer. Two levels are fundamentally an intent-perspective and extent-perspective*^16^.

Observational heterarchy is not only an abstract notion but also a computational model. The model is the time-state-scale re-entrant system (TSSRS) (Gunji et al., 2008; Sasai and Gunji, 2008) consisting of two perspectives: one is a logical self-reference paradox derived from an external observer (Figure 2C); the other is a frame-problem derived from an internal observer (Figure 2D). The logical self-reference paradox is a mixture of levels, whole of system (time-scale) and subsystem levels (state-scale). In a dynamical system, behavior of a system is expressed as a time development of its state. However, the state is obtainable only from the system's boundary condition in which only the upper level, a theorist, can provide. An operation of developing the state of the system (time development: time-scale) and that of providing the state (boundary condition: state-scale) is independent. TSSRS make the two operations re-entrant and invalidate the self-reference paradox (Figure 2C). The invalidation provides re-framing of the system by changing boundary conditions that mean invalidation of the frame-problem (Figure 2D).

**Figure 4:**
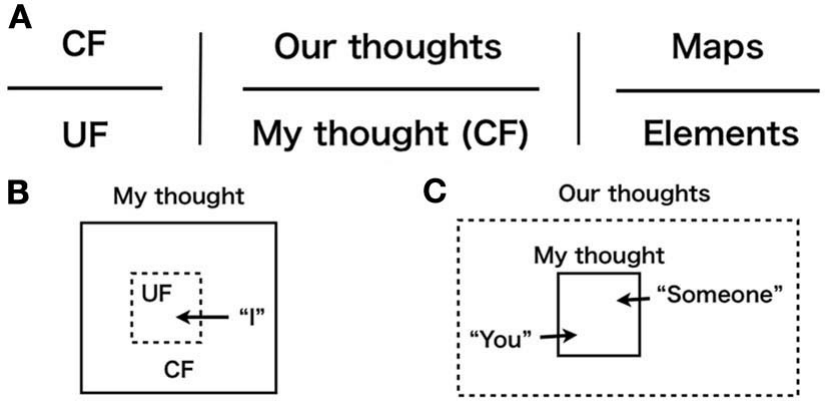
**(A)** Assumed hierarchies on which observational heterarchy is based in this paper: abstract brain activity (left), All thought category in mental processes (middle), and Sets category (right). **(B)** Observational heterarchy with a compression effect in thought category: “I operate on me” (usual agency). **(C)** Observational heterarchy with an extension effect in the thought category: “You operate on me” or “Someone operates on me.”

In observational heterarchy, mediation of the self-reference paradox (a mixture of levels) provides re-framing of hierarchical structures, compression effect (Figure 2C) and an extension effect (Figure 2D) (we will define these notions in the next section). Here, it is noteworthy that we can identify a re-framing in the nature of agency (Wegner, 2002; Wegner et al., 2004). Consequently, there must be a mediating process of an apparent mixture of levels, observational heterarchy, in the nature of agency. Now, for discussions, we defined some hierarchical structures in light of agency. Figure 4A presents three structures in which upper components correspond to upper levels: CF interprets UF, our thoughts include my thought, and maps are applicable to elements^17^. Figures 4B,C show re-framing phenomena in the observational heterarchy: “*I*” operates on me (Figure 4B), and “*you*” or “*someone*” operates on me (Figure 4C). In the next section, we explain the application of observational heterarchy to a mental causal path (Wegner and Wheatley, 1999; Wegner, 2003) and resolve it.

## Observational mental causal process

**Figure 5:**
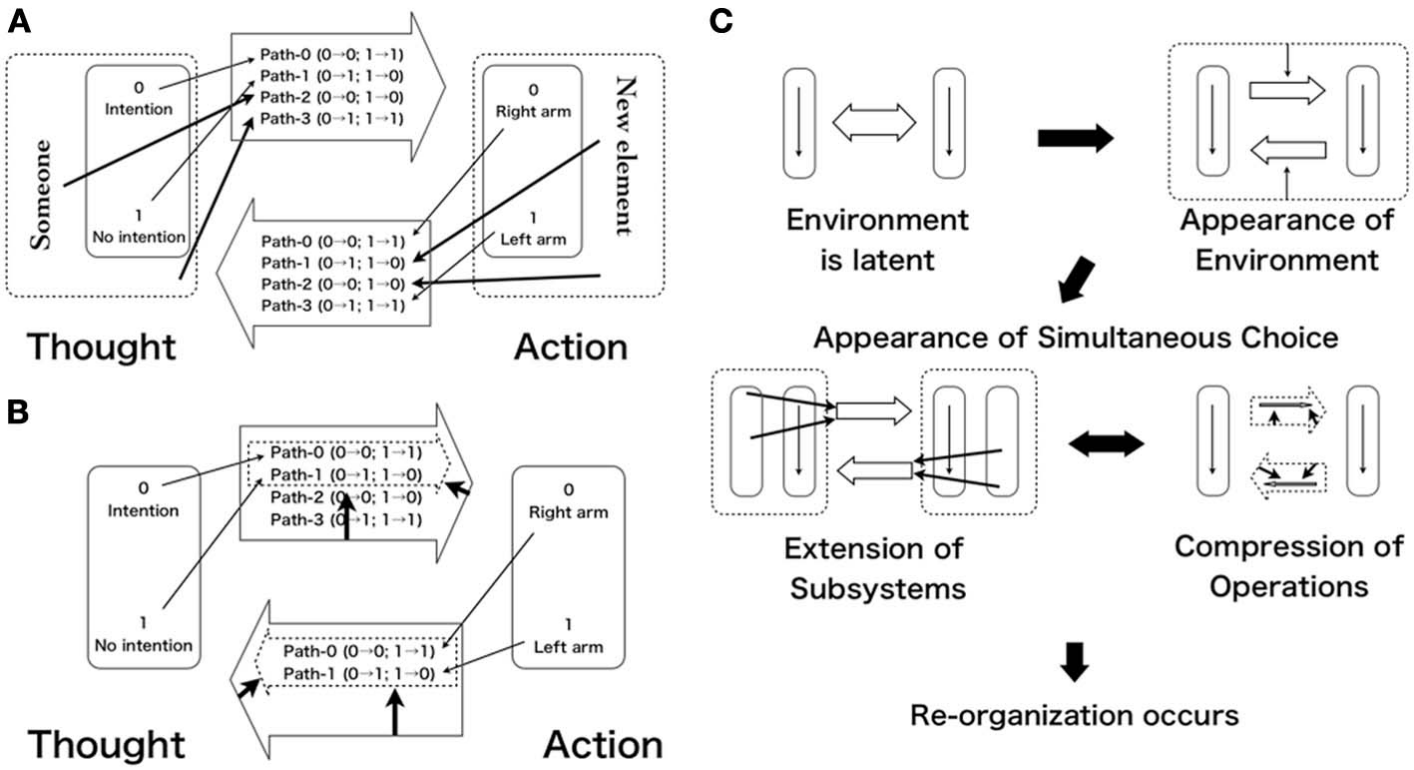
**(A)** Extension of thought/action set. **(B)** Compression of path set. **(C)** Development of observational heterarchy.

A mental causal path can be formalized as follows. In a case of body movement, “thought” is an intention to move and “action” is an objective movement (Figure 5A) (movement usually consists of a pair of a body part and a content of the movement, but now the body part represents the pair). Executing a movement means mapping an intention to a body part (right arm) that is 0 (intention) → 0 (right arm) (Figure 5A). Simultaneous choice can be found in the mixture between thought and the mental causal path (mapping of thought–action). It is unavoidable that one realizes movement of a particular body part and not moving other parts simultaneously when one executes a movement. That means raising the right hand while not raising the left hand. We do not simply raise the right hand without keeping the left hand to balance our posture when raising the right hand. In other words, we cannot separate mapping an intention to a body part from mapping no intention to other parts. However, several mapping exist, we must consider all combinations between each element of a thought set and each of the action set (Details are in the next section). In other words, we choose one path (mapping) from the path set concurrently with choosing an intention (element) from the thought set. In sum, we conduct some kind of logically impossible operation by simultaneously choosing an element at lower level and mapping at a higher level (In usual computations, the element is substituted into prepared mapping after selected). This operation corresponds with simultaneous choice in observational heterarchy.

Following the summary of observational heterarchy (1)–(5) presented above, we summarize the application specifically (Figures 5A–C).

1. Define a set of value for the thought and the action as St = {0(intention), 1(not intention)}, and Sa = {0(right arm), 1(left arm)}, respectively. We designate all possible operations from the thought to the action Path-0, -1, -2, and -3, in the set of the mental causal path. Operations are defined as follows.
  Path-0: 0 → 0; 1 → 0,Path-1: 0 → 1; 1 → 1,
  Path-2: 0 → 0; 1 → 1,Path-3: 0 → 1; 1 → 0.
  Then, we obtain the path set as Sp = {Path-0, Path-1, Path-2, Path-3}.
2. Assuming a mixture of different levels (sets of the thought and path), these two sets are mutually identified. Consequently, one-to-one correspondence is needed. That requires a surjective map from the thought set to the path set^18^. (Because the converse case is clearly possible, we omit that case here).
3. One-to-one correspondence between these two sets involves a logical collapse because the two sets differ in size (path set is a power set of thought set). Therefore, the thought set is smaller than the path set because the former has only two elements but the latter has four elements. For instance, if we map 0 to path-0 and 1 to path-1, respectively, no element exists in the thought set that can map to path-2 or path-3 (Figure 5A). Then the difference in size leads to the impossibility of one-to-one correspondence. Logical collapse can be inferred from the simultaneous choice (the mixture of different levels).
4. However, re-organization through a mediation process should occur here. Specifically for sets of thought and path, two solutions exist: one is extension of the thought set and the other is compression of the path set^19^. In a case of extension, a set can be re-organized by adding two other elements (someone) from out of the set (Figure 5A). Results show that the value in the thought set changes from {0, 1} to {0, 1, 2 (someone), 3}. Conversely in a case of compression, the path set of two elements can be reconstructed by reducing path-2 and path-3 from the original set of four elements. Consequently, this set of two corresponds with the thought set of two (Figure 5B). This solution is temporary. Therefore, reconstruction of elements and maps should occur after resolving it.
5. As described above, observational heterarchy is not an actual entity but something observed by internal measurement. Therefore, sets of thought and action have intent-perspective and extent- perspective, similarly to internal (cause) and external (effect) descriptions in behavior. Consequently, the mental causal path can be resolved by application of the observational heterarchy model.

We present a development of the mental causal path as an observational heterarchy (Figure 5C). Usually, we can act as we intend to. A pair of thought–action as a mental causal path is realized here. In other words, intent–perspective is consistent with extent–perspective as a behavior. However, this assertion of consistency is merely an approximation. Simultaneous choices between intra-level and inter-level are latent in such a normal condition. It appears under abnormal conditions in experiments.

The apparent mixture of different hierarchical levels can be shown in the problem of self-referential mixture between the thought set and path set. As described at the beginning, however, the system never collapses despite some kind of self-referential condition. The mixture results in a collapse in logic, but not in the living systems. The body (system) never engenders collapse but engenders one-to-one correspondence by making consistence. This feature is called robustness. That means to engender one-to-one correspondence can be regarded as reconstruction of a set (frame). Such a reconstruction cause can be formally interpreted as two aspects, compression and extension of a set. As described below, we suggest that these two aspects of mediation of one-to-one correspondence correspond, respectively, with prediction and postdiction. Postdiction can be understood as the aspect of extension effect like a rubber-hand illusion (e.g., Botvinick and Cohen, 1998; Tsakiris and Haggard, 2005), out-of-body experience (e.g., Blanke and Mohr, 2005; Lenggenhager et al., 2007) or embodiment of instruments (e.g., Iriki et al., 1996; Maravita and Iriki, 2004; Sonoda et al., 2012). However, prediction can be understood as the aspect of a compression effect that compresses various interpretations related to cause set attenuation of sensation. Details will be described later. In sum, postdiction and prediction are not problems of the comparison mechanisms, but are instead derived from the perceptual difference in mediation of conflict between CF and UF as to agency.

## Postdiction and prediction

When we devote attention to experimental data of postdiction, we can find the extension process that specific experimental conditions cause unexpected feeling for observers. For instance, alien hand (e.g., Banks et al., 1989; Wegner, 2002; Biran and Chatterjee, 2004) or table turning (Wegner, 2002) are feelings of being moved by someone unknown. They can just arise for actors with thought extension. These examples show extension of the thought set (SoA). The following are examples of extension as to the action set [Sense of Ownership (SoO)]. The I-spy study (Wegner and Wheatley, 1999) or vicarious agency experiment (Wegner et al., 2004) shows the illusion of agency by which a subject feels SoA despite not operating by him in fact. These phenomena are regarded as illusions in the attribution of intention. Thereby they can be regarded as extension actions because a subject's attribution of their intention to action by others means that they choose elements from outside of the action set. Thus, it can be regarded as extension of SoO to some extent. This corresponds with the case in which a new element appears as presented in Figure 5A. The aspects will correspond with extension of SoO as reported by Botvinick and Cohen (1998) and by Lenggenhager et al. (2007). Regarding visual awareness, Eagleman and Sejnowski (2000) reported the perception of a ring trajectory despite its absence in fact. As described above, it is also regarded as extension effect.

In a case of prediction, the compression process can be identified. We can observe it in the experiment reported by Bays et al. (2006). Attenuation of the sensation was observed by self-generated tactile means. In brief, this observation indicates that sensation by touch becomes weaker when one touches one's own hand by oneself than when touched by others. Bays et al. (2006) constructed an apparatus consisting of a torque motor to realize two conditions: self-generated tactile (contact trial) and non self-generated tactile conditions (no-contact and delay trial). In the apparatus, when the right finger presses the button, the torque begins to rotate, resulting in the left finger being pressed (pulse). They differentiated self-generated tactile conditions with non-self-generated one by manipulating the duration between the time of button press and that of torque rotation in milliseconds. Therefore, without delay, it becomes a self-generated condition even though the torque intermediates (contact trial). With delay, it becomes a non-self-generated condition (delay trial). It becomes a no-contact condition if the button is out of alignment. At the moment if a sensor device senses the finger movement and it actuates the motor and presses left finger, the same finger movement can cause a pulse (no-contact trial). In the no-delay condition, when a subject's finger contacts the button (contact trial) that is a self-generated tactile condition. But, whekin a subject's finger does not contact the button (no-contact trial), which means a non-self-generated tactile in the sense of postdiction. Note that attenuation of sensation is observed in the self-generated tactile condition. However, identical results were shown not only in the contact trial but also in the no-contact trial (Experiment 1 in Table 2). Therefore, it was concluded that attenuation of sensation was not postdictive but predictive.

**Table 2:** **Summary of results of Bays et al. (2006)**.

	**Pretrail**	**Posttrail**	**Attenuation**
Experiment 1	Contact	Delay	×
		No-contact	○
Experiment 2	No	Delay	×
		No-contact	×

**Figure 6:**
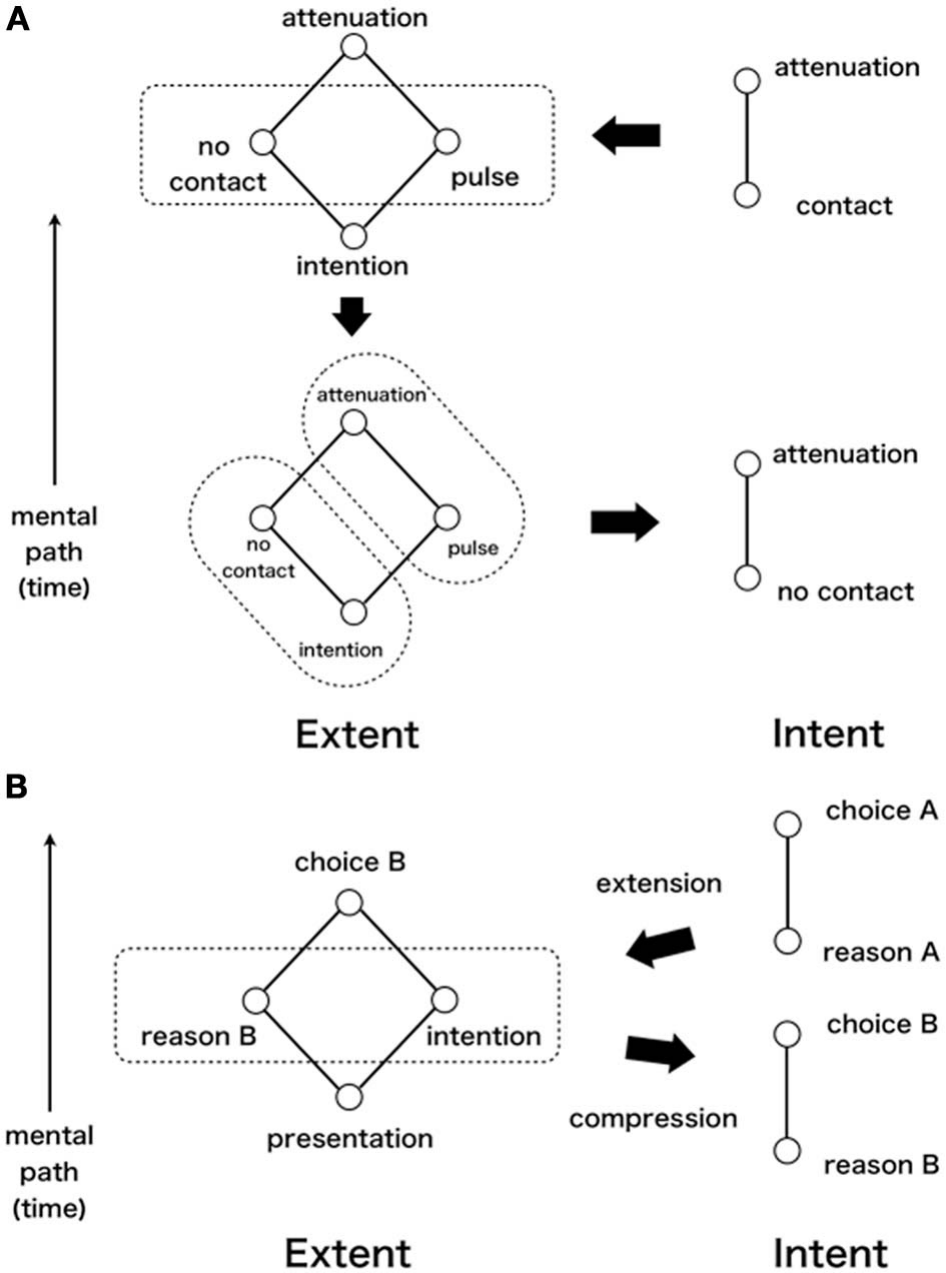
**(A)** Process of attenuation of sensation. **(B)** Process of choice blindness.

Note the assumption that the difference between contact and non-contact is discriminated after the button press event. Then the fact of attenuation despite the discrimination indicates that this perception is not postdictive but predictive. The problem here is the assumption of discrimination after the event. Although the discrimination indicates whether it is self-generated or not by contact, it is the problem that the discrimination and the pulse are perceptually in synchrony. How to address this synchronicity is a problem. In other words, we can find the problem of how we can interpret a causality of button press and pulse in the experimental setting (upper left side in Figure 6A). What we should devote attention to here be the fact that the compression process of interpretation, the contact trial, was regarded as the same trial as the non-contact trial. Moreover we should confirm the result that attenuation of sensation (interpretation of self-generated tactile) was not observed in the no-contact trial without a contact trial in the other experiment of Bays et al. (2006) (Experiment 2 in Table 2).

Following Gunji and Kamiura (2004), we try to describe this situation with division into internal description (Intent) based on subjective report and external one (Extent) based on orders of objective events^20^. In this description, we use lattice structure (Davey and Priestley, 2002) and an order relation is a mental path (time) such as event A-event B when event A occurs before event B^21^. In the contact trial, both Intent and Extent became the contact–attenuation order, mental path (time) shown in Figure 6A (upper right side). In the no-contact trial, however, Extent became a partial order set where, for simultaneous feeling of no contact and pulse, they have no order relation, as shown in Figure 6A (upper left side). At this moment, the order of intention–attenuation might be readily apparent. No contact and pulse were arranged between them. In other words, because there were obvious order relations such as intention—no-contact and intention—pulse, these relations should be described as the lattice structure depicted in Figure 6A (upper left side). Considering mapping to Intent, several interpretations exist (groupings)^22^. Because contact and intention were trained by repetition, no contact and intention would be grouped in Extent (lower left side in Figure 6A). Intent of no-contact—attenuation would be formed (lower right side in Figure 6A). In a no-contact trial, appearance of contradiction between no contact and pulse as to the order relation can trigger the compression of interpretation. We can consider the compression as derived from by repetition in contact trial because the finger movements of no-contact trial are same as those of contact trial (Experiment 1 in Table 2). Note that, in the advance contact trial, grouping between contact and intention is not so readily apparent but trained. In fact, even in the contact trial, attenuation can never be observed with delay. Alternatively, in the no-contact trial, attenuation is not observed either without contact trial (Experiment 2 in Table 2).

Are postdiction and prediction mutually independent effects? No. They must be just concurrent effects, but different in their own ways. Specifically they play different roles in the re-organization of sensation and perception. We explain this in a choice blindness experiment (Johansson et al., 2005). If the experimenter changed a picture that a subject had chosen to another one in secret, then subjects made up a reason for why they chose it even though they actually did not choose it. Again we try to describe this situation with division into Intent and Extent. In Intent, before changing to the other picture, order was reason A – choice A (upper right side in Figure 6B). After the change, it became reason B – choice B (lower right side in Figure 6B). In Extent, after the change, representing a fake picture and fake result of choice were sure (presentation B – choice B), whereas reason B and intention were unsure (left side in Figure 6B). By compression playing a role of prediction, reason B – choice B were reflected in Intent (right pointing arrow). However, it was impossible without producing reason B by extension: a role of postdiction (left pointing arrow). Consequently, it is concluded that postdiction and prediction emerge as the difference of two aspects of extension and compression in the organization of causality, even in the experimentally manipulated contradictive situation. Therefore, we are always internal observers. When perceiving a world that we cannot supervise, our perceptions necessarily accompany both postdiction and prediction.

## Applications of our frameworks to experimental paradigms

Our frameworks of awareness will be testable within some experimental paradigms, based on a gap or mixture of different sensational/perceptional information (intent-/extent-perspective). Specifically, they predict re-framing of thought/action set in a mental causal path (Table 3)^23^. SoA and SoO will be corresponded to a thought and action set, respectively. Their dynamical duality relation (re-framing of the sets) can be derived from our frameworks naturally. Although there are some discussions about a relation between SoA and SoO (e.g., Gallagher, 2000; Tsakiris et al., 2006), they cannot predict such re-framings comprehensively. Additionally, ours can derive out of body experience (OBE) (Blanke and Mohr, 2005) and sleep paralysis (Santomauro and French, 2009) jointly while the other frameworks do not even mention their relation. In our frameworks, OBE and sleep paralysis is corresponded to extreme version of extension effect and compression one for re-framing of an action set (SoO), respectively^24^.

**Table 3:** **Experimental paradigms derived from our framework**.

	**Intention**		**Action**	
	**Normal**	**Extreme**	**Normal**	**Extreme**
Compression	Ouija board	Automatism	Disownership	Sleep paralysis
Extension	Hypnotism	Group will	Embodiment	Out of body experience

For the re-framing of thought sets, one feels oneself operated by someone (hypnotism), group will, something like a ghost (Ouija board), or nothing (automatism) (Wegner, 2002)^25^. For the re-framing of action sets i.e., cognitive body frame, disownership (de Vignemont, 2011) and embodiment (Botvinick and Cohen, 1998) are famous and our frameworks also predict sleep paralysis and OBE (Table 3). Although there are eight experimental paradigms from our prediction, six out of eight have been already established and herein we only show the rest of them, sleep paralysis and OBE. Our frameworks correspond to the below experimental paradigms: extension effect—OBE, and compression effect—sleep paralysis.

### Out-of-body experience (test of extension effect)

A particular subjective sensation called “out-of-body experience (OBE)” was reported (e.g., Blanke and Mohr, 2005; Ehrsson, 2007; Lenggenhager et al., 2007). Their procedures were based on mixture between visual and haptic information through a head mount display (HD) that showed subject's own back touched by a stick in real time. In this section, we introduce a new preliminary construction (Gunji et al., 2013) that causes a feeling of OBE, which differs from that of the previous studies. They used the system of substituted reality (SR) (Suzuki et al., 2012). Their experimental design is based on mixture of subjective and objective view. This design matches with our frameworks.

The SR system consists of multiple video cameras, recorder, and HD. In their design of OBE, a subject sitting in a room wears a helmet-type HD equipped with a subject-eye camera. He first sees an experimenter in front of him with naked eye, and after wearing HD he sees subjective viewed scene via HD. After that, the scene recorded by the objective eye cameras set in front of him is projected in HD. The subjective view and objective view are exclusive with each other, although they are both sides of the same coin—“now.” They cannot be united by a single event in this situation. However, if he experiences continuous change between objective and subjective cameras, he can feel that he himself exists in his own subjective view. In a preliminary experiment, a subject can feel OBE in the situation. That is not just an experience in which a subject can see himself. He can feel that he creates objective view as if it was his lucid dream. Therefore, in this feeling exclusive subjective and objective scenes are united as a single event, different from the feeling experienced in the previous studies.

The mixture of subjective and objective scene leads the integration of the two scenes, and a subject gets objective view in which he can see himself. Then, an expanding “*self*” who has objective view, out of the body, appears. Note that self is a relation of the world and me. This paradigm of OBE demands to switch from the concept that self is reliable to the new one that self is flexible. This is the self who can expand itself in our frameworks. The paradigm of materializing the gap of subjective and objective view as ones lucid dream may also give an understanding of depersonalization disorder (Lambert et al., 2002).

### Sleep paralysis (test of compression effect)

Sleep paralysis is a consciously experienced paralysis either when going to sleep or waking up. During an episode, one is fully conscious, able to open ones eyes but aware that it is not possible to move limbs, head or trunk (Dahlitz and Parkes, 1993; Santomauro and French, 2009). Sleep paralysis can be considered to be an intrusion of rapid eye movement (REM) sleep characteristics into wakefulness. That is, the muscles of the body are deeply relaxed and they cannot be moved with ease, and the dreamlike element with hallucinations may result from the brain activity “dreaming” that is typical of this sleep period (Dement and Kleitman, 1957). Putting it simply, there is a gap between the conscious activity in the brain and the deeply relaxing body: the gap may cause the unmovable body with consciousness. Note that one can move his relaxing body in normal sleep and cannot in sleep paralysis. Consequently, here is the self who compresses oneself into the unmovable body, a compressed self. This self contrasts to that of OBE.

There is currently no known way to induce sleep-onset REM periods, which have been found to be associated with sleep paralysis (Santomauro and French, 2009). But, note that the SR systems can cause a feeling of a lucid dream to some extent by continuous changing between subjective and objective view. Although there is no evidence, the SR system could cause a sleep paralysis like experience. It would need careful designs. One of them may be a continuous change between a real time scene (he see his moving body) and a recorded one (he sees not moving body), which causes some degrees of a gap between intention to move and resultant movement like in sleep paralysis. A subject could mediate the gap by not moving his body with a feeling in his lucid dream. If we could develop these methods, they might have an effect to retain behaviors to some extent. Consequently, with these methods, we could apply them to retrain behavioral disorders such as hyperactivity disorder for rehabilitations. These applications may be contrast to a “mirror box” that can cause movement of unmovable phantom limbs (Ramachandran and Rogers-Ramachandran, 1996).

## Conclusion

A self-referential problem of mixture between different levels (element and map) can be mediated in two processes: compression and extension of a system. However, we should not regard them simply as different effects at the same level. We do not consider observational heterarchy simply as the model that can account for both postdiction and prediction, considering the fact that element and map have originally different status. Mediation of compression is compression of map, which means that one maintains the attitude that “the world is just what I predicted” even if inconsistency exists in the map (interpretation). However, mediation of extension is extension of an element, which means the de-construction of the frame of self for inconsistency. Compression is a transcendental viewpoint that enforces institutionalization from the outside, whereas extension is an internal measurement that intends to make some adjustment from the inside. These two aspects are different levels in the mediation process. In this sense, prediction and postdiction are not mechanisms for the event (a normal feeling of doing is not fundamental because we can feel it even if without an efferent copy), but rather represent difference in the aspect of mediation. Consider the situation in which conscious will and unconscious will come together and an inconsistency appears. Prediction is the aspect that conscious will maintains the process persistently. Then “I” equal “the *other* in my brain.” Conversely postdiction is the aspect by which conscious will is threatened and enforced by unconscious will to adjust. Then the gap separating “I” and “the *other* in my brain” is materialized as “someone.”

Consequently, awareness can be found in such a conflict between conscious will (CF) and unconscious will (UF) that engender origin of voluntariness. It should be identified as a process having duality in the sense that it opens the world (postdiction) and that it closes (prediction).

### Conflict of interest statement

The authors declare that the research was conducted in the absence of any commercial or financial relationships that could be construed as a potential conflict of interest.

## Footnotes

^1^In this study, we use CF and UF in the abstract sense.

^2^Many discussions based on the comparator model (Frith et al., 2000b) invariably presume an author list, {*I, Michael, Cathy*, …}. Furthermore, we choose only one of the authors from the fundamental list depending on our situations. We sometimes mistake a choice. For example, we choose “*Michael*” instead of “*I*.” This paradigm shows that agency is only a mechanism with error or illusion. However, *self* and *others* must be completely different categories. If that were not true, then the subject/object problem would disappear. Furthermore, the problem is in subject itself. The distinction of subject and object will be corresponded to that of CF and UF. Awareness involves the problem in itself. Our argument is that this problem can be formalized using set theory. The author list can correspond to a set of natural numbers {*1, 2, 3, …*}. Then we can place “arbitrary *n*” in the set where “*n*” is obtained after we survey the infinite set, although we actually cannot. “*n*” actually points nothing in the set. If we choose “*1*,” “*1*” is a negation of the others (*2, 3*, …) in the set. However, “*n*” is a denial of the set itself. Thus, “*n*” is completely different status from the other numbers. Consequently, the placement of “*n*” into the set is a category mistake, but we do it easily in mathematics. Herein the “*n*” is “*I*” itself. The sentence “*the other is I*” represents a category mistake. However, agency is beyond the logical mistake. Therefore, we can state that the agency of “*I*” is emergence. In other words, “*n*” represents latency and “*1*,” possibility: “*n*” is changeable after chosen since it is a meaningless sign and “*1*” is not changeable. We discuss the details of this argument below in the text.

^3^The original category of “*I*” is extended to “the other is *I*.” In other words, {*I*} is extended to {*the other, I*}.

^4^Our approach may be close to Bitbol's interventionist-constitutive view (Bitbol, 2012). Body is not fundamental but an observable (i.e., cognitive boundary) for an observer even if it is his own body. Mind is the same case. We concern about an observational process (an internal observer) for an observed relation of some kind of two levels (e.g., body and mind), and what the problem is if we admit that a substance can be such an observer. In the notion of internal measurement (Matsuno, 1989; Gunji, 1993, 2006), we express the relation as a mathematical duality and weaken it by various ways for the use in science. This dynamical duality can be an expression for the latency of downward causation.

^5^Some current discussions distinguish compositional hierarchy and subsumption hierarchy (Salthe, 2012).

^6^POS (Davey and Priestley, 2002) is defined as the following. If an element and an order are expressed as an alphabet and ≤, respectively, POS satisfies (1) *a* ≤ *a*, (2) *a* ≤ *b* and *b* ≤ *a* imply *a* = *b*, (3) *a* ≤ *b* and *b* ≤ *c* imply *a* ≤ *c*. The growth mode of hierarchy (Salthe, 2012) can be expressed as application of order-homomorphism between the POSs. It should be observational heterarchy or contradictory (heterarchy) if not the case.

^7^Herein, the expression of the “wholeness” does not mean the whole of the observer's focal level. It means the whole of all of every level in a hierarchical system. By contrast, in a usual sense, “outside” and “inside” are defined, respectively, as upper levels for the focal level and lower ones. Furthermore, an observer in the focal level cannot know about the outsides and insides (Salthe, 2012).

^8^Gunji (2006) point out three aspects of generation—origin, norm, and variation—by weaving the concepts of Deleuze and Guattari (1991), *plan d'immanence* and *les personnages conceptuels*, and his own considerations into his original theory of life, *weak wholeness, the meditating term, and internal observer*. Herein, we refer to the concepts of “wholeness” discussed in Gunji (2006).

^9^A pair of intent and extent can define the Classification concept. Intent is an attribute of a concept. Extent is a collection of objects or specified models to which the concept is applicable. If we observe “cheese” as a concept, then its intent is “a food derived from milk” and its extent is a collection of “Mozzarella, Parmigiano-Reggiano, Ricotta cheese, … ” The pair of intent and extent can also define a concept of a set. The intent of a set of even numbers is “2N where *N* is a natural number.” The extent of the set is “2, 4, 6, …”

^10^Try to consider the extent of wholeness = the world. The definition that extent is a collection of objects to which a concept is applicable forces to us out of the concept. However, we cannot observe out of wholeness = the world. Here we can identify the impossibility of defining the extent of wholeness = the world. “Possible world” presents us with the same case.

^11^Russell's paradox is the following (Whitehead and Russell, 1925). First define class 1 set as a set that does not include itself (itself is not one of its elements). Next define class 2 set as a set that includes itself. For example, English is class 2 because it includes “English.” Japanese is class 1 because it does not include “Japanese” (this word is in English). From this distinction, we can classify every set as class 1 or class 2. Here we make *M* by collecting all of class 1 sets. *M* does not include itself if *M* is class 1. However, the definition of *M* means that *M*, a collection of all of class 1 sets, includes itself because *M* is class 1. Therefore, it ends up in a contradiction. Next, when we assume that *M* is class 2, *M* includes *M*. However, from the definition of *M*, *M* does not include *M*. This also presents a contradiction. Finally, *M* cannot be class1 or class 2. This explanation shows characteristics of the Russell's paradox.

^12^From the definition of a concept, a pair of intent and extent defines a set dually. We can express the intent of a set as *y* = {*x* | *A*(*x*)} if we define *A*(*x*) as a nature of *x*. Extent of the set *y* is *x* ∈ *y* if *y* is a set. Equivalence of intent and extent is ∃*y*∀*x* (*x* ∈ *y* ⇔ *A*(*x*)). Russell's paradox can be derived easily from this notation. The definition of the class 1 expresses *A*(*x*) as *x* ∉ *x*. From the equivalence of intent and extent, we can obtain *x* ∈ y ⇔ x ∉ x. Therein, *x* is arbitrary and *y* is special. Therefore, we can exchange *x* by *y* and obtain *y* ∈ *y* ⇔ *y* ∉ *y*. This is Russell's paradox. The paradox derives from the mixture of elements and sets. Current set theories forbid mixture by adding the restriction that element *x* is one element of arbitrary set *a*. Set theories define the restriction as separation schema ∀*a*∃*y*∀*x*(*x* ∈ *y* ⇔ *x* ∈ *a* ∧ *A*(*x*)).

^13^In Russell's paradox, we first assumed that we could check all sets and divide them into two types of sets according to whether a set can include itself (class 2) or not (class 1). This assumption engenders a contradiction in Russell's argument. We mean this assumption as looking over a description of sets.

^14^In Cantor's diagonal argument (Moore, 1991), Cantor used the argument to extend the notion of a cardinal number. The argument only shows a contradiction of a statement for which the size of an infinite set *S* and that of the power set of *S* (the set of all partial sets of *S*) are the same. However, this negative argument led to the new limit of infinity (countable infinity) in mathematics. We herein identify a positive creation from the negative argument in the working of mathematicians. Gunji (2006) reconsidered a meaning of the diagonal argument to clarify the concept of internal observer who bears a positive meaning of negation (the notion of weak wholeness). In the argument, two kinds of “wholeness” of all of infinite bit strings are identified. “Intent-wholeness” is defined by some kind of counting operation ({*1, 2, 3*,…}) and “extent-wholeness” by its use ({…, *n*, …}) for a comparison with an inverted diagonal bit string. Roughly speaking, intent-wholeness is a set of all of infinite strings before the comparison of each of all strings in the argument. Extent-wholeness is one after the comparison. Therefore, we define “weak wholeness” as a notion that is intermediate of intent-wholeness and extent-wholeness. For example, Cantor creates the new limit of infinity (countable infinity) for an intermediate of them from the diagonal argument. Consequently, observational heterarchy with weak wholeness has intent- and extent-perspectives explicitly (Gunji and Kamiura, 2003, 2004).

^15^In this quote, “*heterarchy*” means observational heterarchy.

^16^In the usual case, Intent and Extent are defined when a concept is being given. However, we generalize it here and consider the Intent and Extent that give a concept. We refer the definition by Gunji and Kamiura (2003) below. Definition (Generalized Intent and Extent): Given a concept, Intent is defined as a collection of attributes of the concept, and Extent is defined as a collection of objects to which the concept is applied. Conversely, given two collections of attributes and objects, if each object has all attributes and each attribute contributes to all objects, a pair of collections is called a pair of Intent and Extent. Then we say that Intent and Extent constitutes a concept. The operations by which an attribute in Intent is applied to an object in Extent are called inter-level operations. A triplet, <Intent, Extent, inter-level operation> constitutes a concept.

^17^From Libet et al. (1983), RP precedes conscious intention. In our description, UF precedes CF. We can say that UF converted actions and that CF interprets the actions. This sketch can also be used to identify aspects of the comparator model (Frith et al., 2000b). Interpretation indicates an order relation (Salthe, 2012). Therefore, CF is higher than UF. However, we mean some kind of order relation in which a mixture of levels is latent.

^18^Simultaneous choice is the definition of mixture of different levels and that expressed as a mapping. Mapping is defined as an operation from domain to codomain. Domain and codomain can be regarded as having different status, such as a start point and a target. Mixtures of different levels mean those of these two. The set defining a map is required domain and codomain dually. Furthermore, the map is not unidirectional but bidirectional, and then it also has nature of duality. Considering these requirements, isomorphic (bijective) mapping is a necessary and sufficient condition: Because mappings for all elements must be defined according to the requirement that a domain is a codomain, a surjective map is needed. If it is not injective map, it becomes one to many mapping in the opposite direction. Thereby an injective map is needed. As a result of the use of a surjective map and injective map needs, a bijective map is required.

^19^Gunji and Kamiura (2003, 2004) emphasized only the extension effect in re-organization derived from a mixture in observational heterarchy. This paper, however, indicates also a compression effect as a result of careful consideration of the mixed situation. We reconsider the difference between these two aspects as the difference between postdiction and prediction.

^20^McTaggart (1989), a philosopher, proposed a model for subjective and/or cognitive time. He evaluated two kinds of models, called A series and B series. The B series consist of events linearly ordered, and is designed by “before” and “after,” on one hand. The A series consists of past, present and future which cannot co-exist and exclusive with each other, on the other hand. This original pair of the A and B series is utilized as a causal set (Bombelli et al., 1987) and its semantics in the field of quantum mechanics (Markopoulou, 2000), independent of philosophy. Gunji et al. (2009) studied a relation or interaction of the two series. In that study, both the series are defined as lattices (Davey and Priestley, 2002).

^21^We show the definition of causal set, partially ordered set (POS), and lattice, in brief. A causal set consist of separable events. Each event can be connected by another event via a directed edge without loops. If two events are connected by two edges that have different directions, they are equivalent to each other. Thus, these particular directed networks can be expressed as a POS (Davey and Priestley, 2002). If an event and directed edge are expressed as an alphabet and ≤, respectively, POS satisfies (1) *a* ≤ *a*, (2) *a* ≤ *b* and *b* ≤ *a* imply *a* = *b*, (3) *a* ≤ *b* and *b* ≤ *c* imply *a* ≤ *c*. For lattice, we also add some terminologies. Any elements *a* and *b* in a POS, *P*, are anti-chain with each other if neither *a* ≤ *b* nor *b* ≤ *a* does not hold. For any subsets *Q* ⊆ *P*, join of *Q*, denoted by ∨ *Q* is defined by such that for any *q* ∈ *Q*, *q* ≤ ∨ *Q* and if *q* ≤ *s*, then ∨ *Q* ≤ *s*. Especially, if *Q* is a two elements set such as {*a*, *b*}, ∨ {*a*, *b*} is represented by *a* ∧ *b*. Similarly meet of *Q*, denoted by ∧ *Q* is defined by such that for any *q* ∈ *Q*, *q* ≥ ∧ *Q* and if *q* ≥ *s*, then ∧ *Q* ≥ *s*. Especially, if *Q* is a two elements set such as {*a*, *b*}, ∧ {*a*, *b*} is represented by *a* ∧ *b*. Given a partially order set, *P*, if for any *x*, *y* ∈ *P*, *x* ∧ *y*, *x* ∨ *y* ∈ *P*, then *P* is called a lattice. For example, a four elements lattice {*a, b, c, d*} such as that in Figure 6A (left side) has order relations {*a* ≤ *b*, *a* ≤ *c*, *b* ≤ *d*, *c* ≤ *d*, *b* and *c* are anti-chain}.

^22^Considering lattice-homomorphism from Extent to Intent, there are four: (1) {all elements} → {attenuation}, (2) {all elements} → {no-contact}, (3) {no contact, attenuation} → {attenuation}; {intention, pulse} → {no-contact} (4) {no contact, intention} → {no-contact}; {attenuation, pulse} → {attenuation}.

^23^Although we described a compression effect for interpretations (maps) in the discussion presented above, we can also presume the effect for thought/action sets (elements) according to a mediation process, e.g. in a case of an extreme compression for maps.

^24^In both OBE and sleep paralysis, one is fully awareness. Thus, agency seems to be unchanged. Ownership associated with cognitive body would be changed to some kind of a compressed (unmovable) body in sleep paralysis and an extended (out of) body in OBE. These extreme offsets of SoO may cause an abnormal body feeling.

^25^Although this classification is expedient, we think that it is suggestive. In the classification, Ouija board is expressed as a compression effect since one may feel ghost's agency and its presence is not strong. Automatism is the same case. By the way, we can also assume that an extreme concentration in sports as an example of automatism instead of questionable studies of automatism (Wegner, 2002).
